# Heart Rate Variability as an Alternative Indicator for Identifying Cardiac Iron Status in Non-Transfusion Dependent Thalassemia Patients

**DOI:** 10.1371/journal.pone.0130837

**Published:** 2015-06-17

**Authors:** Karn Wijarnpreecha, Natthaphat Siri-Angkul, Krekwit Shinlapawittayatorn, Pimlak Charoenkwan, Suchaya Silvilairat, Chate Siwasomboon, Pannee Visarutratna, Somdet Srichairatanakool, Adisak Tantiworawit, Arintaya Phrommintikul, Siriporn C. Chattipakorn, Nipon Chattipakorn

**Affiliations:** 1 Cardiac Electrophysiology Research and Training Center, Faculty of Medicine, Chiang Mai University, Chiang Mai, Thailand; 2 Cardiac Electrophysiology Unit, Department of Physiology, Faculty of Medicine, Chiang Mai University, Chiang Mai, Thailand; 3 Center of Excellence in Cardiac Electrophysiology, Chiang Mai University, Chiang Mai, Thailand; 4 Department of Pediatrics, Faculty of Medicine, Chiang Mai University, Chiang Mai, Thailand; 5 Department of Radiology, Faculty of Medicine, Chiang Mai University, Chiang Mai, Thailand; 6 Department of Biochemistry, Faculty of Medicine, Chiang Mai University, Chiang Mai, Thailand; 7 Department of Internal Medicine, Faculty of Medicine, Chiang Mai University, Chiang Mai, Thailand; 8 Department of Oral Biology and Diagnostic Sciences, Faculty of Dentistry, Chiang Mai University, Chiang Mai, Thailand; Northeastern University, UNITED STATES

## Abstract

**Background:**

Iron-overload cardiomyopathy is a major cause of death in thalassemia patients due to the lack of an early detection strategy. Although cardiac magnetic resonance (CMR) T2* is used for early detection of cardiac iron accumulation, its availability is limited. Heart rate variability (HRV) has been used to evaluate cardiac autonomic function and found to be depressed in thalassemia. However, its direct correlation with cardiac iron accumulation has never been investigated. We investigated whether HRV can be used as an alternative indicator for early identification of cardiac iron deposition in thalassemia patients.

**Methods:**

Ninety-nine non-transfusion dependent thalassemia patients (23.00 (17.00, 32.75) years, 35 male) were enrolled. The correlation between HRV recorded using 24-hour Holter monitoring and non-transferrin bound iron (NTBI), hemoglobin (Hb), serum ferritin, LV ejection fraction (LVEF), and CMR-T2* were determined.

**Results:**

The median NTBI value was 3.15 (1.11, 6.59) μM. Both time and frequency domains of HRV showed a significant correlation with the NTBI level, supporting HRV as a marker of iron overload. Moreover, the LF/HF ratio showed a significant correlation with CMR-T2* with the receiver operating characteristic (ROC) curve of 0.684±0.063, suggesting that it could represent the cardiac iron deposit in thalassemia patients. HRV was also significantly correlated with serum ferritin and Hb.

**Conclusions:**

This novel finding regarding the correlation between HRV and CMR-T2* indicates that HRV could be a potential marker in identifying early cardiac iron deposition prior to the development of LV dysfunction, and may be used as an alternative to CMR-T2* for screening cardiac iron status in thalassemia patients.

## Introduction

Thalassemia intermedia are mild forms of thalassemia and known as non-transfusion dependent thalassemia (NTDT) [1, 2]. Anemia in NTDT is typically not as severe as thalassemia major or transfusion dependent thalassemia (TDT). However, heart failure from iron overload cardiomyopathy is still the most common cause of death in NTDT patients despite the effectiveness of iron chelating agents [3, 4]. Iron-overload in NTDT patients is caused by an increased gastrointestinal iron absorption secondary to chronic anemia, ineffective erythropoiesis, and hepcidin deficiency [3–9]. The increased iron absorption could lead to the accumulation of iron at a rate of 2–5 grams per year depending on the degree of bone marrow expansion and peripheral hemolysis [7, 10]. Eventually, NTDT patients develop iron overload leading to serious complications as seen in the TDT patients, complication including end-organ damage due to iron overload, bone changes and hypercoagulability [7, 8, 10]. Currently, not only the prevention of iron overload, but early detection of iron overload cardiomyopathy is also an important strategy to prevent cardiac failure in NTDT patients.

It has been shown that iron overload cardiomyopathy can be reversible only if early intensive chelation therapy has been initiated [11–13]. Over the past decades, several biochemical and clinical parameters as well as radiological investigations have been used to determine the cardiac iron status. Serum ferritin has been widely used for decades to represent the iron status in the body due to its strong correlation with liver iron concentration [12, 14]. However, it is not just specific to the iron-overload condition [12, 15, 16], and low levels of serum ferritin do not necessarily represent a low risk of iron overload cardiomyopathy [17]. Non-transferrin-bound iron (NTBI), a low molecular weight and free-form iron, can be detected in plasma when serum transferrin is fully saturated [18, 19]. This form of iron can cause cardiovascular injury and cardiac cell apoptosis by its reduction and oxidation of free iron [3, 5, 18, 20–27]. Several studies demonstrated that NTBI levels can be used as an index to indicate iron-overload conditions and also have a significant correlation with the vital organ damage in TDT [28–31] and NTDT [32, 33] patients. As a result, NTBI can be one of the best biochemical parameters that represent cardiac iron status. However, their use is limited due to the cost, and it is not available in all research centers. Although the cardiac magnetic resonance (CMR) T2* is currently used as a gold standard for early detection of iron overload cardiomyopathy [5, 17, 34], its use is currently limited due to its cost and availability worldwide. According to this limitation, CMR T2* might not be the best practical strategy for early detection of iron overload cardiomyopathy or screening the cardiac iron status in thalassemia, especially for those in developing countries. Other available tests, such as conventional electrocardiogram, endomyocardial and liver biopsy, and echocardiography are not shown to be helpful for early detection of iron overload cardiomyopathy [12, 35–37]. In contrast, growing evidence suggests that heart rate variability (HRV), an easily accessible diagnostic parametric, reliable, low-cost and non-invasive test can represent cardiac autonomic function, and could be an important practical assessment strategy for early detection of iron overload cardiomyopathy in thalassemia patients [12, 38, 39].

For decades, HRV has been used to evaluate cardiac autonomic function in patients with heart disease, including post-myocardial infarction [40–42], heart failure, and also TDT patients who are prone to have iron overload cardiomyopathy and increased risk of heart failure [38, 40, 43–45]. Although previous studies demonstrated the depressed HRV status in thalassemia, there is no study showing the correlation between HRV and CMR T2* in NTDT patients. In this study, we sought to determine whether HRV can be used as an alternative indicator for early detection of the cardiac iron status in the thalassemia patients. The hypotheses were tested that HRV has a strong correlation with the NTBI and also CMR T2*. In this cross-sectional study, HRV was determined in NTDT patients, and the relationship between HRV and biochemical parameters, clinical parameters, echocardiographic findings, and CMR T2* values were evaluated.

## Materials and Methods

### Study protocol

The study protocol was approved by the Institutional Ethics Committee of the Faculty of Medicine, Chiang Mai University, Chiang Mai, Thailand. NTDT patients enrolled in this study were defined as having thalassemia and receiving no more than seven RBC transfusions per year in the last five years, with age ranged between 10–50 years, and did not have any infectious diseases or acute illnesses during the investigation. Exclusion criteria included patients with a contraindication to MRI, and clinical evidence of other secondary causes of pulmonary hypertension, including human immunodeficiency virus infection, hepatitis virus infection, collagen vascular diseases, cirrhosis, chronic obstructive airway diseases and acquired heart disease associated with pulmonary hypertension. All patients were treated at Chiang Mai University Hospital during September 2013 to May 2014. All patients and their parents in the case of minorities gave written informed consent.

Patient charts were reviewed for thalassemia diagnosis and genotypes, transfusion history, history of splenectomy, details of drugs history, and comorbidities. The body weight, height and liver size were recorded in each patient. Laboratory profiles (Mean Hb and serum ferritin in the last 12 months, hematocrit (Hct), maximum ferritin in the past 5 years, plasma NTBI, labile plasma iron (LPI)), echocardiography, 24-h Holter ECG recording for HRV analysis, CMR T2* were collected and analyzed at the time of entry. The correlation between HRV and NTBI, hemoglobin (Hb), serum ferritin, LV ejection fraction (LVEF), CMR T2* were determined.

### Heart rate variability measurement

The SEER Light Holter system (GE Healthcare, Milwaukee, WI, USA) was used. An ECG was recorded continuously for 24 h. The computer software MARS software version 7, GE Healthcare, Milwaukee, WI, USA was used to scan for rhythm disturbance and to detect and label each QRS complex. Excessive noise and artifacts were noted, and ectopy was quantified. The time-domain analyses included average heart rate, average R-R intervals (NN), standard deviation of the R-R intervals over a 24-h period (SDNN), standard deviation of all 5-min mean R-R intervals (SDANN), average standard deviation of all 5-min R-R intervals (ASDNN), the percentage of R-R intervals with more than 50-ms variation (pNN50), and the square root of mean squared differences of successive R-R intervals (rMSSD). The frequency-domain analyses were done with the same analytical software using Fast-Fourier transform analysis. The obtained frequency-domain indices were the total power (0–0.4 Hz), high-frequency power (HF, 0.15–0.4 Hz) spectral density, low-frequency power (LF, 0.04–0.15 Hz) spectral density, and very-low-frequency power (0.003–0.04 Hz) spectral density. Total power expresses the magnitude of the entire HRV, whereas HF power reflects the parasympathetic tone, and LF power indicates the sympathovagal interactions[40]. These power spectral densities were expressed in absolute units (ms^2^).

The designated physician who operated and fitted the Holter monitor to the patients was blinded to the patients’ information. The subjects were fitted with the Holter monitor after blood sample collection at the Holter unit of the Faculty of Medicine, Chiang Mai University. In the present study, none of the included patients took medications such as beta-blockers, calcium channel blockers, statins, and ACE-inhibitors which could affect the HRV.

### Laboratory profiles

Mean Hb and serum ferritin in the last 12 months, hematocrit (Hct), maximum ferritin in the past 5 years, plasma NTBI, labile plasma iron (LPI) were determined in each patient at the date of entry.

### Measurement of hematological parameters

Complete blood counts were performed using an automated hematological analyzer (Beckman Coulter A^C^.T 5diff, Coulter Corp., Miami, FL, USA).

### Measurement of ferritin

The Roche Cobas E601 (Germany) Immunology Analyzer was used to determine serum ferritin using a sandwich principle. In the first incubation, a plasma sample of 10 μL, a biotinylated monoclonal ferritin-specific antibody, and a monoclonal ferritin-specific antibody labeled with a ruthenium complex formed a sandwich complex. In the second incubation, after addition of streptavidin-coated microparticles, the complex became bound to the solid phase via the interaction of biotin and streptavidin. The reaction mixture was aspirated into the measuring cell where the micro particles were magnetically captured onto the surface of the electrode. The unbound substance was then removed with ProCell/ProCell M. The application of a voltage to the electrode then induced chemiluminescent emission which was measured by a photomultiplier. Results were determined via a calibration curve which was specifically generated by a 2-point calibration and a master curve provided via the reagent barcode [46, 47].

### Quantification of plasma NTBI

Plasma NTBI was determined as described in the previous study [48]. First, a small volume of aluminium chloride solution (a final concentration of 200 μM) was added to 450-μL plasma and incubated at room temperature for 1 hr. Then, 50 μL of 800-mM nitril-otriacetic acid (NTA) (a final concentration of 80 mM) solution at pH 7.0 was added and the mixture was incubated for 30 minutes at room temperature to produce a ferric-nitrilotriacetate complex, Fe^3+^-(NTA)_2_. Then, the Fe^3+^-(NTA)_2_ was separated from plasma proteins by spinning plasma mixture through a membrane filter (30-kDa cut-off, polysulfone type, 0.5-mL capacity) at 12,000 *g*, 15°C for 45 min. The ultrafiltrate was injected into a non-metallic 50-μL loop and analyzed with the high performance liquid chromatography (HPLC) technique. HPLC requirements were composed of a glass analytical column (ChromSep-ODSI, 100 x 3.0 mm, 5 μm) and a mobile-phase solvent (3 mM CP22 in 19% acetronitrile buffered with 5 mM MOPS pH 7.0). Having been isocratically eluted with the mobile-phase solvent at a flow rate of 1.0 mL/min, NTBI as Fe^3+^-(NTA)2 was fractionated on the column, then immediately on-column derivatized with CP22 to form a Fe^3+^-(CP22)_3_ complex. The resulting orange-colored Fe^3+^-(CP22)_3_ product was on-line detected at 450 nm with the SpecMonitor 2300 flow-cell detector (LDC Milton-Roy Inc., FL, USA). The NTBI peak height was integrated and recorded for further determination of NTBI concentration from the calibration curve. A calibration curve was produced by plotting the obtained peak height values on the *y*-axis against iron concentration on the *x*-axis. An equation of linear regression line was used to calculate the plasma NTBI concentration.

### Measurement of LPI concentration

In principle, redox-active LPI converts dihydrorhodamine (DHR) to oxidized fluorescent rhodamine (R), leading to an increase in fluorescence intensity (FI) [21]. A plasma sample of 20 μL was incubated with/without 5 mmol/L DFP at 37°C for 30 min and DHR solution containing ascorbic acid was then added (180 μL). Kinetic spectrofluorometry (λex 485 nm, λem 538 nm) was performed immediately for 40 min at 37°C with a reading every 2 min. The increasing FI was followed and the slope of the FI was plotted against the reaction time between 15–40 minutes. A calibration curve was created from a standard ferrous ammonium sulfate (FAS) solution (0–20 μmol/L). The difference in the rate of DHR oxidation represents a component of the redox active LPI in plasma. The LPI concentration was calculated from the calibration curve relating to the difference in the slope with/without DFP versus the standard iron concentration [49].

### Echocardiographic studies

The standard two dimensional and Doppler trans-thoracic echocardiography was performed at rest in all enrolled patients with Philips iE33 (Philips Healthcare, Bothell, WA, USA). Left ventricular end-diastolic dimension (LVEDD) and left ventricular end-systolic dimension (LVESD) were measured, and left ventricular ejection fraction (LVEF), stroke volume, cardiac output, and fractional shortening (FS) were determined based on the calculation of LV volumes by the method of discs, following the recommendations by the American Society of Echocardiography (ASE), and using apical two- and four- chamber views to measure cardiac function [50, 51].

Impaired systolic LV dysfunction (LVEF < 55% or LVFS < 35%) was also determined in NTDT patients[51]. Diastolic function was evaluated from mitral inflow velocities (E-wave, A wave, E/A ratio) and deceleration time (DT) using pulsed wave (PW) Doppler in the apical four-chamber view. PW tissue-Doppler velocities were acquired at end-expiration, in the apical four-chamber view, with the sample positioned at the lateral mitral annulus, measuring early diastolic (E’) and late diastolic (A’) velocities and calculating the E/E’ ratio. Diastolic dysfunction was defined by E/A ratio, DT and E/E’ according to the current guideline [52].

### Magnetic resonance imaging

Magnetic resonance acquisition was performed using a 1.5T MR scanner (GE, HDxt, Milwaukee, WI, USA), using an 8-channel body array. For the measurements of myocardial iron overload, we used a T2* gradient—echo multi-echo sequence (flip angle 25 degree, matrix: 192 x 128 pixels, field of view 38 x 28.5 cm, bandwidth 125 kHz, slice thickness 10.0 mm, number of excitations 1, view per segment 4, repetition time 18.5 ms). A single mid ventricular short-axis view of the left ventricle was acquired at eight echo times (TEs) (1.7 ms, which increased in 2.0–2.1 ms increments) in a single end-expiratory breath-hold.

Cardiac magnetic resonance (CMR) T2* analysis was performed on a workstation (Advantage Window 4.6, GE healthcare) using commercial analysis software (StarMap 4.0, GE Healthcare). According to the previous study, the 95% confidence interval of normal CMR T2* value (>20ms) was assessed [17, 53]. The values 14–20 ms were regarded as mild iron overload, values between 10 and 14 ms were regarded as moderate iron overload, and a T2* less than 10 ms was regarded as severe iron overload [17].

### Statistical analysis

Numerical data are presented as median (25th, 75th percentile) due to the skewed distribution of numerical data. Differences between splenectomized and non-splenectomized NTDT patients were performed using the parametric independent student T-test. CMR T2* was regarded as the gold standard diagnostic technique. The correlations between HRV and laboratory profiles, echocardiographic parameters, CMR T2* were determined using the Spearman's rank correlation. A *P* value of less than 0.05 was considered statistically significant. Statistical analysis was performed with the use of SPSS version 13.0 for Windows (SPSS Inc., Chicago, IL, USA). The receiver operative characteristic (ROC) curve was analyzed, and the binary logistic regression analysis, forward likelihood method, was also applied to determine the association between CMR T2* and HRV.

## Results

One hundred patients were enrolled in this study. One patient was excluded since this patient refused the MRI investigation. Clinical and biochemical characteristics, echocardiographic parameters, CMR T2* values, and HRV of NTDT patients (n = 99) were summarized in Table 1. NTDT patients in this study consisted of 62 beta-thalassemia and 37 Hb H diseases (71 had never had a blood transfusion, 12 had blood transfusion only once in the last 12 months, and 16 had blood transfusion < 7 times in the last 12 months). A total of 35 patients (35%) had a splenectomy before the evaluation. Forty-two patients (42%) were receiving iron chelating drugs (24 deferiprone, 3 desferrioxamine, 2 deferaxirox, and 13 combined iron chelating agents).

**Table 1:** Demographic, clinical, biochemical characteristics, echocardiographic measurement, cardiac magnetic resonance (CMR) T2*, time-domain and frequency-domain HRV parameters of non-transfusion dependent thalassemia (NTDT) patients (n = 99).

Parameters	Median (25th, 75th percentile)
Age (years)	23.00 (17.00, 32.75)
***Gender***	
Male (n (%))	35 (35.4%)
Female (n (%))	64 (64.6%)
Splenectomy (%)	35 (35.4%)
Number of blood transfusions in last 12 months	0.00 (0.00, 1.00)
Mean hemoglobin in last 12 months (g/dL)	7.80 (7.10, 8.78)
Hematocrit (%)	25.10 (22.00, 28.80)
MCV	64.90 (59.50, 71.10)
Mean ferritin in last 12 months (ng/dL)	673.50 (318.00, 1009.25)
Maximum ferritin in the past 5 years (ng/dL)	849.00 (396.00, 1925.00)
NTBI (μM)	3.15 (1.11, 6.59)
LPI (μM)	1.12 (0.01, 1.93)
***Echocardiographic values***	
LVEDD (mm)	4.90 (4.50, 5.30)
LVESD (mm)	3.10 (2.88, 3.40)
LVEF (%)	65.80 (61.79, 70.10)
FS (%)	36.20 (32.75, 39.50)
CMR T2* (ms)	43.95 (39.68, 48.75)
***HRV-Time domain***	
SDNN (ms)	114.00 (95.00, 142.00)
SDANN (ms)	105.00 (86.00, 130.00)
ASDNN (ms)	43.00 (37.00, 56.00)
rMSSD (ms)	25.00 (18.00, 35.00)
***HRV-Frequency domain***	
LF (ms2)	14.98 (11.54, 18.35)
HF (ms2)	11.17 (7.66, 16.60)
LF/HF ratio	1.39 (1.16, 1.61)

NTBI, non-transferrin-bound iron; LPI, labile plasma iron; SDNN, standard deviation of all normal sinus R-R intervals in the entire 24-h recording; SDANN, standard deviation of all averaged normal sinus R-R intervals for all 5-min segment in the 24-h recordings; ASDNN, average of the standard deviations of all R-R intervals for all 5-min segments in the 24-h recordings; rMSSD, root mean square of the mean of the squared difference of two consecutive R-R intervals; LF, low frequency power; HF, high frequency power; LF/HF ratio, ratio of power in low/high frequency.

In this study, the median CMR T2* was 43.95 (39.68, 48.75) ms which indicated that the NTDT patients have preserved CMR T2* values (T2* > 20 ms, n = 98). The comparison of HRV between the splenectomized (n = 35) and non-splenectomized (n = 64) group was shown in the supplementary table (S1 Table). Time-domain (SDNN and SDANN) HRV parameters were significantly lower in the splenectomized group (107.00 (81.00, 137.00) vs 122.00 (99.75, 151.00) and 96.00 (69.00, 116.00) vs 115.50 (88.25, 138.75), *P* = 0.008 and 0.002, respectively). Moreover, the number of blood transfusions and serum ferritin in the last 12 months, maximum ferritin in the past 5 years, NTBI, and LPI were also significantly higher in the splenectomized group.

The correlations analysis between time and frequency domains of HRV and clinical and biochemical parameters was presented in Table 2. All time-domain (SDNN, SDANN, ASDNN and rMSSD), and frequency-domain (LF and HF) HRV parameters showed a significant correlation with mean Hb, mean serum ferritin levels in the last 12 months, maximum ferritin in the past 5 years, and the number of blood transfusion in the last 12 months. Moreover, both time-domain (SDNN and SDANN) and frequency-domain (LF/HF ratio) HRV parameters were also showed a strong correlation with the NTBI. However, none of the HRV parameters showed any correlation with echocardiographic parameters or LPI levels (data not shown). Since the echocardiographic data demonstrated that the enrolled patients did not have any abnormal LV function whereas the NTBI level was detected, these findings suggested that the patients were in an early stage of iron overload without any functional abnormality or impaired systolic or diastolic function. Moreover, a significant correlation between HRV parameters and CMR T2* was demonstrated in which the LF/HF ratio showed a significant correlation with CMR T2* values (*R* = -0.227, *P* = 0.026).

**Table 2:** The correlations between HRV parameters and other clinical parameters in non-transfusion dependent thalassemia (NTDT) patients.

Spearman's rho	Mean hemoglobin in last 12 months	Mean ferritin in last 12 months	Maximum ferritin in the past 5 years	NTBI	Number of blood transfusions in last 12 months
***HRV-Time domain***					
*SDNN (ms)*	0.387‡	-0.338‡	-0.484‡	-0.224*	-0.314†
*SDANN (ms)*	0.345‡	-0.332‡	-0.482‡	-0.221*	-0.326‡
*ASDNN (ms)*	0.462‡	-0.364‡	-0.505‡	-0.138	-0.259†
*rMSSD (ms)*	0.330‡	-0.324‡	-0.445‡	-0.187	-0.237*
***HRV-Frequency domain***					
*LF (ms2)*	0.487‡	-0.341‡	-0.474‡	-0.093	-0.230*
*HF (ms2)*	0.408‡	-0.334‡	-0.448‡	-0.169	-0.221*
*LF/HF ratio*	-0.045	0.152	0.196	0.204*	0.094

*P < 0.05.

^†^ *P* < 0.01.

^‡^ *P* < 0.001

NTBI, non-transferrin-bound iron; SDNN, standard deviation of all normal sinus R-R intervals in the entire 24-h recording; SDANN, standard deviation of all averaged normal sinus R-R intervals for all 5-min segment in the 24-h recordings; ASDNN, average of the standard deviations of all R-R intervals for all 5-min segments in the 24-h recordings; rMSSD, root mean square of the mean of the squared difference of two consecutive R-R intervals; LF, low frequency power; HF, high frequency power; LF/HF ratio, ratio of power in low/high frequency.

**Fig 1:**
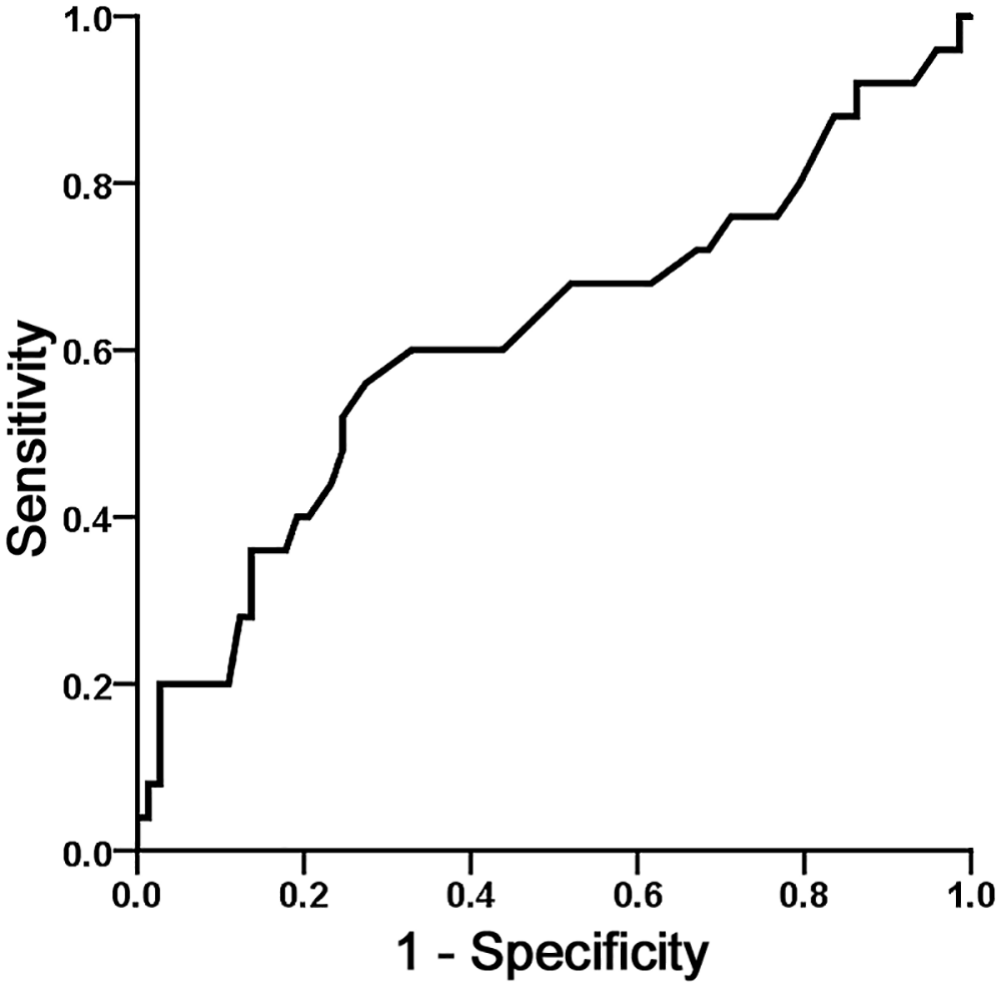
Receiver operating characteristic (ROC) curve of the HRV for the prediction of myocardial iron deposition determined by CMR T2*.

From the correlations analysis between CMR T2* and HRV, and clinical parameters, only LF/HF and mean Hb in last 12 months were correlated with CMR T2* values. The binary logistic regression analysis was performed to determine the correlations of CMR T2* and HRV and other clinical parameters. Using the multivariate forward likelihood method with adjustments for age, sex, splenectomy, and iron chelating drug use, we found that only the LF/HF (odds ratio 8.710, 95% CI 2.324–32.649, *P* = 0.001), but not the mean Hb in the last 12 months significantly correlated with CMR T2*. Since there is only one NTDT patient in this study that had CMR T2* <20 ms, we could not use the same cut point to determine cardiac iron overload as shown previously in TDT patients [17]. Therefore, the lowest quartile CMR T2* value (40 ms) of our study population was used as the cut point to determine cardiac iron overload in NTDT patients. The predicted area under the ROC curve of mean Hb level in last 12 months was 0.618±0.071 (95%CI: 0.478–0.757; *P* = 0.08). However, the predicted area under the ROC curve of LF/HF was 0.684±0.063 (95%CI: 0.561–0.807; *P* = 0.006) (Fig 1).

## Discussion

The major findings in this study are that the time-domain and frequency-domain of HRV parameters showed a significant correlation with the NTBI, mean Hb, serum ferritin, number of blood transfusions, and CMR T2*.

HRV assessment is a technique that measures the beat-to-beat variability in the R-R intervals, which can imply the cardiac autonomic regulation. The decreased time-domain parameters or increased LF/HF ratio indicates there is a cardiac sympathovagal imbalance which is generally known as “depressed HRV” [40, 54]. Depressed HRV has been demonstrated in both thalassemic mice and thalassemia patients, and has been shown to be associated with cardiac autonomic dysfunction and can lead to the early detection of iron overload cardiomyopathy [12, 38, 55, 56]. Despite those reports, there is no clinical study that directly investigates the relationship between HRV and NTBI and CMR T2* to elucidate the potential of HRV as the alternative assessment of cardiac iron status.

Growing evidence from studies on thalassemic mice suggests that they had high NTBI levels and also had depressed HRV compared to the wild-type mice [12, 57, 58]. In the present study, it has been demonstrated for the first time the significantly correlations between HRV parameters and the NTBI, mean Hb, ferritin and number of blood transfusions in the last 12 months, and maximum ferritin in the past 5 years of NTDT patients. These findings emphasize the potential role of HRV as an indicator of iron status under iron overload condition.

It has been proposed that HRV may be used to indicate cardiac iron accumulation. Available data in animal studies supported this possibility by demonstrating that HRV showed a correlation with iron accumulation in the heart [12, 58]. Despite reports in animal studies, the direct correlation between HRV and CMR T2* together with NTBI has never been investigated in a clinical study. Although there is one small clinical study with 34 TDT patients which showed no correlation between HRV and serum ferritin, and NTBI [39], the study itself was too small to find a statistically significant correlation. As demonstrated in the present study, the correlation between HRV and CMR T2* and NTBI suggested the possibility that HRV might be an alternative indicator of both body iron status and cardiac iron status under iron overload conditions. This study also demonstrated for the first time that only LF/HF was significantly correlated with CMR T2* values as determined by the binary logistic regression analysis. ROC curve analysis also demonstrated that only LF/HF was significantly correlated with CMR T2* values. These findings suggested that LF/HF could be the best clinical parameters to predict CMR T2* values for early detection of cardiac iron overload in NTDT patients. Moreover, it has been shown that cardiac autonomic dysfunction develops before myocardial contractility dysfunction or cardiac iron loading in TDT patients [41]. Therefore, CMR T2* and echocardiography, which cannot evaluate cardiac autonomic function, still have limitations when compared to the HRV.

In splenectomized patients, it has been shown that they had significantly higher serum ferritin, transferrin saturation, and desferrioxamine-induced urinary iron excretion (DFU) when compared to the non-splenectomized patients [59]. In the present study, it was found that the NTBI, LPI, and maximum ferritin in the past 5 years were also significantly higher in the splenectomized group. These findings strongly indicated that the spleen plays an important role in iron regulation [59]. The free form of NTBI both in plasma (LPI) and intracellular (labile cellular iron, LCI) has been shown to produce reactive oxygen species (ROS) via Haber-Weiss and Fenton reaction, leading to cardiovascular injury from lipid peroxidation, damage to cellular proteins, nucleic acids, mitochondria, and cellular organelles, and also cardiac cell apoptosis [3, 5, 6, 18, 20–22, 24, 25, 27, 60, 61]. It has been shown that the oxidative stress from many pathological conditions including iron overload can lead to sympathovagal disturbance and significantly depressed HRV [58, 62–70]. This study demonstrated for the first time that the splenectomized NTDT group had a significantly lower time-domain (SDNN and SDANN) HRV compared to the non-splenectomized group. Therefore, it could be suggested that splenectomized patients have higher NTBI and LPI levels leading to oxidative stress-mediated cardiac autonomic dysfunction, thus resulting in depressed HRV as observed in this study.

### Study Limitation

One limitation of this study was the lack of a healthy control group or an additional group with a disease associated with cardiac iron overload, such as hereditary hemochromatosis. Although the inclusion of this latter group would allow us to differentiate whether the effects on HRV are specifically from iron overload, or from a combination of iron overload and anemia, hereditary hemochromatosis is extremely rare in our population, therefore the addition of this group is not possible for this study.

Since most of the severity of cardiac iron overload of NTDT patients in this study were out with the traditional MRI cut-off for significant cardiac iron overload (CMR T2* > 20), we could not divide NTDT patients by a CMR T2* severity assessment. This study performed a cross-sectional study and could not take into account the morbidity and mortality prediction in this group of patients. Moreover, there was a significant female sex bias and the population was predominantly young adults. Both sex and age can influence HRV substantially and may limit the generalizability of the findings. Future prospective studies with long-term follow-up and larger sample are needed for this purpose.

## Conclusion

Early detection of iron overload cardiomyopathy to initiate early chelation therapy in thalassemia patients is a strategy to prevent cardiac complications and decrease the mortality rate. The findings of this study regarding the correlation between HRV and NTBI levels, and CMR T2* indicate that HRV could be a potential marker in identifying body free iron status as well as cardiac iron status in NTDT patients.

## Supporting Information

S1 Table Comparison of HRV indices in non-transfusion dependent thalassemia (NTDT) patients according to splenectomy. (DOC)
