# Supplementary material for: Heart Rate Variability as an Alternative Indicator for Identifying Cardiac Iron Status in Non-Transfusion Dependent Thalassemia Patients
Source: PLoS One. 2015 Jun 17;10(6):e0130837. doi: 10.1371/journal.pone.0130837 (PMC4471165; doi:10.1371/journal.pone.0130837)
Supplement: S1 Table — (DOC) [file pone.0130837.s001.doc]

| **Parameters** |  | **Median**  **(25th, 75th percentile)** | | ***P*-value** |
| --- | --- | --- | --- | --- |
|  | **Splenectomized**  (n = 35) | | **Non-splenectomized**  (n = 64) |  |
| ***HRV-Time domain*** |  | |  |  |
| SDNN (ms) | 107.00  (81.00, 137.00) | | 122.00  (99.75, 151.00) | 0.008 |
| SDANN (ms) | 96.00  (69.00, 116.00) | | 115.50  (88.25, 138.75) | 0.002 |
| ASDNN (ms) | 41.00  (34.00, 51.00) | | 44.00  (38.25, 60.00) | ns |
| rMSSD (ms) | 22.00  (15.00, 35.00) | | 26.50  (20.00, 38.00) | ns |
| ***HRV-Frequency domain*** |  | |  |  |
| LF (ms^2^) | 13.76  (10.69, 17.87) | | 15.53  (11.76, 19.30) | ns |
| HF (ms^2^) | 9.53  (6.37, 13.93) | | 11.65  (8.19, 17.27) | ns |
| LF/HF ratio | 1.44  (1.20, 1.70) | | 1.35  (1.15, 1.56) | ns |
